# Effects of Controlled Oxygen Partial Pressure on Arc Dynamics and Material Erosion in a Pantograph–Catenary System

**DOI:** 10.3390/ma19061234

**Published:** 2026-03-20

**Authors:** Bingquan Li, Zhaoyu Ku, Xuanyu Xing, Ran Ji, Huajun Dong

**Affiliations:** 1 School of Mechanical Engineering, Dalian Jiaotong University, Dalian 116028, China; libingquan@lntdxy.edu.cn (B.L.);; 2 Department of Railway Rolling Stock, Liaoning Railway Vocational and Technical College, Jinzhou 121000, China

**Keywords:** image processing, arc dynamics, pO_2_, arc erosion, current-carrying

## Abstract

Motivated by altitude-induced fluctuations in oxygen partial pressure (pO_2_) and their impacts on PCS off-line arc motion and erosion response, this study proposes a comparative experimental approach featuring single-variable control under constant total pressure and coordinated multi-source electrical-signal observation. A reciprocating current-carrying arc-generation rig was established, in which pO_2_ was equivalently regulated via a constant-pressure gas substitution and mixing approach. High-speed imaging–based quantitative vision analysis was integrated with synchronized voltage–current measurements to evaluate the net effects of five O_2_ volumetric fraction levels (6, 11, 14, 17, and 21 vol%) under a DC supply of 120 V/25 A on arc dynamics, electrochemical processes, and contact pair erosion. Based on repeated-test results, the 14 vol% case exhibited the poorest stability (maximum fluctuation coefficient 20.306%), whereas the 17 vol% case showed the lowest current-carrying efficiency (minimum 56.070%) together with the most severe erosion damage. Moreover, with increasing pO_2_, the erosion morphology evolved in a staged manner, transitioning from localized central ablation accompanied by melt-related traces to adhesive wear-induced delamination, and ultimately to electrochemical oxidative wear. Overall, pO_2_ imposes a pronounced non-monotonic “window effect” on arc stability and erosion, providing key evidence for PCS structural optimization and risk assessment in open operating environments.

## 1. Introduction

The pantograph–catenary system (PCS) is a critical component of railway electrification, enabling efficient power transfer between the overhead line and moving trains. However, transient arcs triggered by vibration-induced separations during relative sliding contact can severely undermine operational stability, leading to localized temperature rise, abrupt degradation of current-collection quality, and accelerated material deterioration of the pantograph strip and contact wire [1,2]. Unlike high-speed railways supplied by AC systems, urban electrified railways that employ rigid catenary and DC traction power are more sensitive to corrugation-induced arcing disturbances [3]. The intense thermal flux generated by these arcs acts on the contact wire surface with an approximately Gaussian spatial distribution, while arc energy can induce internal cracking and material vaporization in the strip, further degrading energy-transfer efficiency [4]. Therefore, elucidating PCS arc dynamics and the associated erosion mechanisms under mechano-electrical coupled multi-physics conditions is a prerequisite for structural optimization and life extension of contact materials.

In recent years, growing evidence has shown that, for PCS contact pairs composed of carbon- and copper-based conductors, wear and erosion are strongly governed by coupled factors such as the ambient environment and material properties [5]. Xu et al. [6] developed a pantograph–catenary arcing platform incorporating a high-speed airflow field and reported that increasing airflow intensifies the longitudinal drift speed and height of the arc column, accompanied by arc-root hopping. Field-tracking tests and current-carrying friction experiments further indicated that reduced humidity accelerates the transition of carbon materials from abrasive wear to oxidation-assisted cracking and substantially modulates the probability of arc energy release [7,8]. Shen et al. [8] also noted that, compared with humidity, temperature fluctuations exert a stronger influence on the friction coefficient and contact resistance of the contact pair. Current-carrying friction tests under extreme temperature conditions concluded that low-temperature operation amplifies both electrical and mechanical wear, thereby deteriorating current-collection quality; conversely, although high-temperature conditions can markedly suppress arc ablation, they also promote the evolution of surface damage from delamination wear to adhesive wear [9,10]. By establishing magnetohydrodynamic (MHD) arc models under low-pressure environments, studies [11,12] identified a critical pressure threshold governing arc stability: when the pressure exceeds this threshold, the stability mode is disrupted and the metal-vapor content as well as thermo-mass transfer effects are enhanced. Oxygen is one of the most critical reactive species at current-carrying tribological interfaces [13], and variations in its partial pressure may jointly affect arc stability and material degradation through plasma sustaining processes and interfacial oxidation. Accordingly, considerable attention has been devoted to the involvement of oxygen in discharge processes and wear mechanisms. Rolling-friction tests reported in Refs. [14,15] showed that the interfacial friction coefficient of carbon-based materials varies systematically with oxygen availability, and that the wear amount exhibits a non-monotonic evolution. More recently, work focusing on current-carrying friction under coupled atmospheres employed multiple characterization techniques to reveal composite oxidation mechanisms in mixed oxygen–water–vapor environments, including thermal oxidation, tribo-oxidation, and anodic oxidation [16]. In summary, these studies highlight the complex effects of environmental factors on the electrochemical behavior of materials, yet they often cannot disentangle the fluid-discharge coupling introduced by changes in total pressure. In particular, systematic investigations that control pO_2_ as a single variable while synchronously linking arc dynamics with erosion response remain relatively limited.

Motivated by engineering scenarios where altitude differences lead to pronounced variations in pO_2_, this study equivalently regulated the O_2_ volume fraction in a sealed chamber using an ambient-pressure gas-exchange mixing method to isolate fluid–discharge coupling effects induced by changes in total pressure, thereby elucidating the net effect of pO_2_ on PCS arc dynamics and erosion mechanisms. An innovatively designed reciprocating current-carrying arc-generation platform is employed to establish a sealed test environment with controllable pO_2_. By integrating digital image processing with multi-source electrical signal acquisition, the full current-carrying frictional discharge process of a “Cu/C” contact pair is captured, and the effects of pO_2_ on arc morphology evolution, arc-root motion characteristics, and current-carrying discharge performance are clarified. Together with micro-morphological analysis of arc-eroded carbon specimens, the mechanism by which pO_2_ governs arc-induced material erosion under current-carrying conditions is elucidated. The results reveal a non-monotonic, stage-dependent modulation of arc stability by oxygen partial pressure and indicate that, within an intermediate-oxygen window, current-carrying quality deteriorates most severely. These findings provide experimental evidence for mitigating contact pair erosion and extending safe, stable service life, and further offer data support for PCS structural optimization and design of electrified railways operating across different altitude regions.

## 2. Experimental Methodology

### 2.1. Experimental Materials

**Figure 1:**
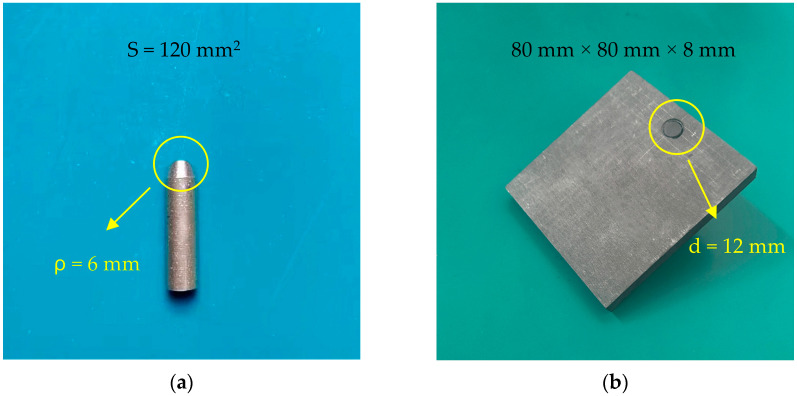
The contact pair specimens. Adapted from Ref. [17]. (**a**) Pure copper rod (Cu-CATH-2); (**b**) pure carbon plate.

All contact pair specimens used in this experiment were manufactured from the same production batch and custom-machined to identical specifications in-house by the institutional workshop at Jinzhou, China. The copper specimen was fabricated from high-purity electrolytic tough pitch copper (Cu-CATH-2) and, following the geometric requirements of the contact-wire profile, was machined into a copper rod with a cross-sectional area (S) of approximately 120 mm^2^ and a tip radius of curvature ρ≈6 mm. The carbon specimen contained no less than 98% carbon and was prepared as a square plate 80 mm×80 mm×8 mm. To ensure a reliable mechanical and electrical connection, a threaded hole with a diameter d≈12 mm was machined at the upper part of the carbon plate, into which a 25 mm-long carbon rod of the same material was embedded. Figure 1 shows the contact pair specimens employed in this study. The two specimens were mounted separately in two insulating epoxy-resin plates, which were further fixed on two vertically stacked dual-rail linear stages, thereby forming a vertically opposed configuration between the specimens.

The detailed physical properties of the contact pair specimens are listed in Table 1.

### 2.2. Experimental Platform

To investigate the regulation mechanisms of pO_2_ on arc-sustaining behavior and arc-induced erosion and wear in the PCS, an environment-controllable dynamic diagnostic platform was established. The platform comprised a power circuit and a measurement-and-control circuit, and its main components included a programmable DC power supply, an adjustable load resistor, a sealed arc-generation chamber, a gas-substitution module, a data-acquisition (DAQ) module, and a high-speed camera. The programmable DC power supply (IT6018D-1500-40, ITECH, Nanjing, China) provides a maximum output voltage of 1500 VDC with an accuracy better than 0.02%, and the circuit current was regulated by the adjustable load resistor to meet the required electrical conditions.

In existing PCS arc studies, an arc-fault generator (AFG) is commonly used to initiate arcs by separating a movable electrode from a fixed electrode along a single separation path [18]. On this basis, the present platform incorporated a redesigned arc-generation device tailored to this study. Specifically, two non-magnetic dual-rail linear stages were arranged orthogonally and driven by stepper motors under a programmable controller, enabling two-dimensional (lateral and longitudinal) relative sliding displacement and independent adjustment of sliding speed. Constrained by dual limit switches, the two opposing electrodes mounted on the stages executed relative motion to reproduce a zigzag sliding trajectory representative of PCS operation. All components were integrated into the arc chamber, whose inner surfaces were lined with a PET thermal-insulation film (light transmittance λ ≤ 5%) to reduce external interference and improve optical observation conditions.

A four-way valve with a G1/2 (BSPP, ISO 228-1 [19]) externally threaded (Swagelok FST Co., Ltd., Shanghai, China) was installed on the chamber top to precisely control the internal gas environment. The upper port was connected to a vacuum pressure gauge (range: −0.1 to 0.15 MPa) to provide real-time feedback on chamber pressure; the right port was connected to high-purity N_2_/O_2_ supplies equipped with a pressure regulator and a mass flow meter for quantitatively controlled gas injection; the left port was connected to an oil-free vacuum pump with a pre-filter to actively evacuate the original gas; and the lower port was inserted vertically into the chamber top as the main gas-flow channel. To ensure gas tightness, all connections were sealed with polytetrafluoroethylene (PTFE) tape, and manual short-handle ball valves were installed upstream of both the left and right ports. A dynamic gas-substitution procedure consisting of “mixing–calibration–steady state” was implemented to reproducibly establish the target pO_2_ cases, thereby providing a stable, controllable, and repeatable atmosphere for the experiments.

The DAQ module was designed to acquire, condition, and store the required analog signals, including circuit voltage, circuit current, ambient temperature and humidity, ambient oxygen concentration, temperature in the arc region, and the contact pressure between the electrodes. The sensor suite included a Hall-effect voltage sensor, a Hall-effect current sensor, a temperature–humidity sensor, an oxygen sensor, a K-type thermocouple, and a pressure sensor. All channels were sampled online at high frequency using a multifunction DAQ device, with a maximum sampling rate of 1 MSa/s. Notably, some channels required signal conditioning via integrated circuits before interfacing with the measurement-and-control circuit, and the detailed models and specifications of the sensors and DAQ device are listed in Table 2.

To suppress resistance-induced interference from other wiring and components in the power circuit, the voltage-sensor leads were connected at the roots of the two contact pair electrodes, and the current sensor was installed on the cathode side close to the test chamber. Study [20] confirmed a significant negative correlation between ambient relative humidity and the electrical conductivity of Cu/C electrode materials. Accordingly, an adjustable electrode humidifier was incorporated to maintain stable temperature and humidity inside the test chamber, and the conditions were synchronously monitored by a temperature–humidity sensor mounted on the chamber wall. This arrangement effectively isolates the influence of ambient thermo-hygrometric variations on current-carrying ablation of the Cu/C pair. Similarly, the oxygen sensor was wall-mounted to monitor the chamber pO_2_ case. Notably, a two-point calibration was performed prior to installation. High-purity nitrogen was used for zero calibration, and a reference oxygen mixture prepared by mass flow controller (MFC) blending was used for span calibration, with calibration coefficients recorded after the reading stabilized. In addition, two temperature-compensated K-type thermocouples were mounted in parallel on the carbon specimen to provide auxiliary feedback on the energy-accumulation efficiency of the electrode material. A thin-film pressure sensor was employed in conjunction with the stepper motor to ensure stable initial contact between the specimens.

**Figure 2:**
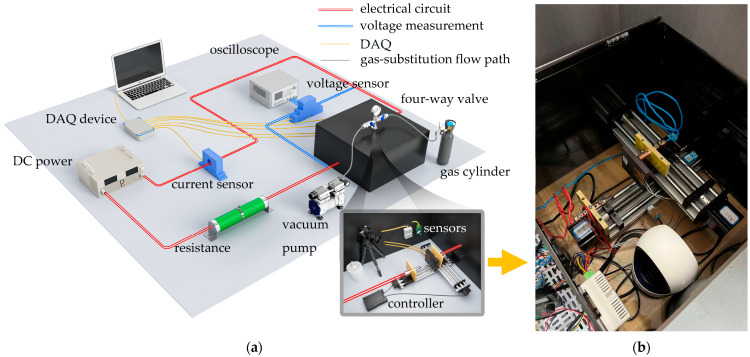
The schematic of the experiment apparatus. Modified from Ref. [17]. (**a**) Electrical circuit, gas-substitution flow path and DAQ; (**b**) arc generation chamber.

The platform also incorporated a high-speed camera to record the entire arc-discharge process under different pO_2_ levels in real time, and a polarizing filter was installed in front of the lens to attenuate harmful radiation generated during arcing. The frame rate was set to 1250 fps, and the image resolution was 720 pixels (px) × 720 pixels (px). Notably, to align the video timestamps with the DAQ timestamps, a low-voltage auxiliary signal line was designed to simultaneously transmit the experiment-start trigger to the shutter control of the high-speed camera. Figure 2 shows the experimental platform developed in this study.

### 2.3. Experimental Conditions

To ensure that all experiments were conducted under consistent environmental conditions, a unified “standard atmospheric condition” was adopted, with the temperature maintained at 23.0 ± 0.3 °C and the relative humidity controlled at 50 ± 2% RH. This standard has been widely used in various engineering tests and serves as a reference for experimental comparability [21]. In the free atmosphere within the troposphere, the molar fraction of oxygen in dry air is approximately 20.95%, whereas that of nitrogen is approximately 78.08%, and both remain nearly constant [22]. According to the standard atmosphere model, the pressure–altitude relationship can be expressed as:

(1) $$P\left(h\right)=P_{0}{\left(1-\frac{Lh}{T_{0}}\right)}^{\frac{g}{RL}},$$

The symbols in the equation are defined as follows:

- *h*: geometric altitude (m);
- *T*_0_: standard sea-level temperature (288.15 K);
- *L*: temperature lapse rate (0.0065 K/m);
- *P*(*h*): total atmospheric pressure at altitude *h* (kPa);
- *P*_0_: standard sea-level atmospheric pressure (101.325 kPa);
- *R*: specific gas constant for dry air (287.05 J/(kg·K));
- *g*: gravitational acceleration (9.80665 m/s^2^).

It can be seen that the total atmospheric pressure in free air is negatively correlated with altitude. Notably, according to Dalton’s law of partial pressures, pO_2_ is jointly determined by the total pressure and the molar fraction of oxygen, and is positively correlated with both [23]. Therefore, as altitude increases, pO_2_ decreases markedly; at an altitude of 4000 m, the oxygen availability is only about 60% of that at sea level [24]. Considering that water vapor occupies a partial pressure in humid air, pO_2_ was humidity-corrected in this study for a rigorous definition, and the corresponding calculation is given by:

(2) $$p_{O_{2}}=x_{O_{2}}\;\left(P\left(h\right)-p_{H_{2}O}\right),$$

(3) $$p_{H_{2}O}=\varphi \cdot p_{ws}\left(T\right),$$

Here, $x_{O_{2}}$ denotes the dry-basis volumetric fraction of oxygen (approximately equal to the molar fraction), $p_{H_{2}O}$ is the partial pressure of water vapor, φ is the relative humidity (0 to 1), and $p_{ws}(T)$ is the saturation vapor pressure at temperature *T*. In this study, pressure is expressed in kPa and *T* is in °C. The Buck empirical equation was adopted to calculate $p_{ws}(T)$, which can be expressed as follows [25]:

(4) $$p_{ws}\left(T\right)=0.61121exp\left[\left(18.678-\frac{T}{234.5}\right)\left(\frac{T}{257.14+T}\right)\right],$$

When temperature and relative humidity are held constant, the above formulation allows the mixed-gas volumetric fraction to be directly converted into the humidity-corrected pO_2_.

This study defined the test cases by anchoring pO_2_ and denoted the test groups using O_2_ volumetric fraction ($x_{O_{2}}$). During the experiments, the total pressure was maintained approximately constant at near-ambient conditions, which can be expressed as:

(5) $$P\left(h\right)=P_{0}\pm 0.5\;kPa,$$

Different pO_2_ test cases were obtained by adjusting the volumetric fractions of O_2_ and N_2_ in the gas mixture. To anchor the experimental boundary cases, a representative high-altitude railway line was used as a reference for equivalent-altitude mapping. The Qinghai–Tibet Railway reaches its highest elevation at Tanggula Pass, at 5072 m above sea level [26]. Accordingly, after humidity correction, four primary test groups were defined in this study: 21 vol%, 17 vol%, 14 vol%, and 11 vol%, corresponding to mapped representative altitude nodes of sea level, approximately 2 km, approximately 3.5 km, and approximately 5 km, respectively. In addition, a 6 vol% case was introduced as an extended extreme group to probe the trend boundary and potential mechanistic transitions. The detailed grouping of test cases is summarized in Table 3.

Bruni et al. [27] reported that the unstable zigzag sliding of the overhead contact line (OCL) induced by lateral excitation can markedly increase the arc initiation frequency. Accordingly, the motion path of the contact pair was strictly constrained in this study, and limit switches were used to fix the starting position and trajectory so as to minimize the coupled influence of sliding errors on the ablation mechanism. Based on multiple rounds of preliminary tests, the lateral sliding speed of the movable-side electrode was set to 10 mm/s, and the longitudinal offline speed was set to 10 mm/s, in accordance with the repeatability requirement. The detailed experimental parameters are listed in Table 4.

Before each test, the surfaces of the contact pair electrodes were ground flat and cleaned to obtain a smooth and consistent initial contact interface. A positive power-supply configuration was adopted, in which the copper specimen served as the anode and the carbon specimen served as the cathode in the electrical circuit. Study [28] has demonstrated that, compared with the negative-feeding mode in traction systems, this configuration can effectively suppress transient phenomena in the arc column. The electrode clamping position in the epoxy-resin holder was fine-tuned to ensure that, prior to each test, the two electrodes were in the same relative contact position. An initial normal load of 50 N was applied using a step controller, and the contact quality was verified with a multimeter by ensuring that the contact resistance satisfied *R* ≤ 10 mΩ, thereby bringing the electrodes into a steady-conduction state ready for testing. To reduce subsequent data-processing uncertainty, the intrinsic resistance of the contact pair electrodes was measured and recorded under static conditions, and the mean value of three measurements was used for experimental consistency verification.

To establish the target pO_2_ test cases under a constant total pressure, an atmosphere-preparation procedure of “evacuation–purging–gas mixing–steady state” was implemented. By switching the ball valves of the four-way valve, the chamber was first evacuated through the vacuum-pump line to a preset low pressure below 10 kPa, and then refilled through the gas-supply line with high-purity nitrogen to restore near-atmospheric pressure. This evacuation–refill cycle was repeated several times to reduce residual oxygen and to unify the initial condition. Subsequently, sequential metered gas filling was performed using an MFC. The chamber pO_2_ was adjusted to the target level according to the prescribed flow-rate ratio, and the total pressure was maintained at the preset value via make-up gas addition or minor venting. After gas mixing, the ball valves were closed and the chamber was sealed and left undisturbed for approximately 3 h to allow sufficient diffusion and homogenization. During this period, $x_{O_{2}}$, total pressure, temperature and humidity were continuously recorded. If the values deviated from the target, a small-dose sequential make-up filling was applied for correction until the online deviation entered the tolerance band and the condition remained stable for more than 10 min. Gas samples were then collected at both the inlet and outlet ports of the chamber and verified by gas chromatography (GC). The experiment was initiated only when the absolute deviation of oxygen volume fraction from the preset value was less than 0.2 vol% and the inlet–outlet difference was within the GC measurement uncertainty.

Study [29], focusing on DC discharges within 75 to 300 V and 6 to 30 A, reported from extensive repeatability tests that the influence of voltage on arc contact resistance is relatively limited, whereas the load current is more sensitive in governing arc characteristics. Data comparisons further indicated that a load current of 25 A enables sustained arcing with relatively stable volt–ampere behavior. Accordingly, once the pO_2_ steady-state condition was established, the adjustable load resistor was tuned so that the DC power supply output was 120 V/25 A. After the circuit was energized and stabilized, the dual stepper motors were driven according to the prescribed motion parameters to reproduce PCS sliding and offline separation for arc initiation. Immediately after arc extinction, the power supply was switched off, the ablation morphology was documented and qualitative indicators (e.g., ablation pits and the heat-affected zone (HAZ)) were marked. The specimens were then sealed for subsequent micro-morphological characterization, and the sensor signals and high-speed imaging data were archived.

With all other experimental parameters unchanged, each single pO_2_ test case was repeated three times. A minimum interval of 30 min was maintained between consecutive runs to ensure sufficient cooling of the electrodes. Before each repeat, the electrode surfaces were re-polished to remove residual oxide layers and ablation traces, thereby mitigating history effects on subsequent discharge behavior and erosion outcomes.

## 3. Results and Discussion

### 3.1. Arc Morphological Evolution and Motion Characteristics

**Figure 3:**
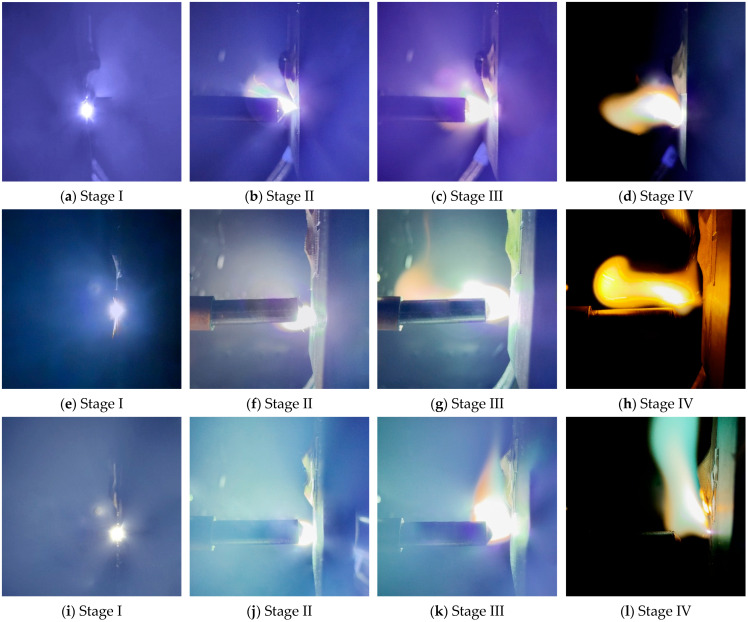

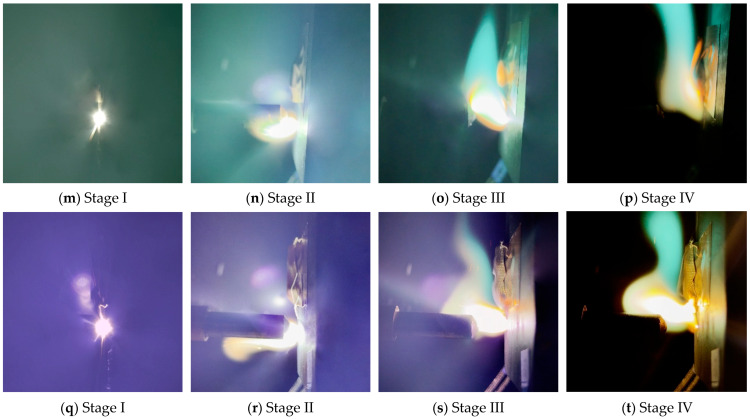
Representative high-speed frame sets illustrating arc evolution under five pO_2_ levels. (**a**–**d**) 6 vol%: Stages I–IV; (**e**–**h**) 11 vol%: Stages I–IV; (**i**–**l**) 14 vol%: Stages I–IV; (**m**–**p**) 17 vol%: Stages I–IV; (**q**–**t**) 21 vol%: Stages I–IV.

The development and evolution of PCS arcs exhibit a distinct stage-wise behavior and generally proceed through four successive stages: arc breakdown, arc expansion, steady arcing, and arc extinction [30]. As shown in Figure 3, after repeated tests under the experimental parameters listed in Table 4, representative frame sets acquired by the high-speed camera were selected for different test cases in each stage based on arc evolution and electrical fluctuation characteristics. Specifically, to avoid subjective bias, representative frames were selected by first defining stage-wise time windows from synchronized electrical signals and then prioritizing the median-time frame within each stage, supplemented by a frame capturing the characteristic morphology of that stage. The same selection strategy was applied to all test cases to ensure comparability. During the arc breakdown stage (Stage I), the current exhibited the first pronounced drop from the pre-arc electrical steady-state level, accompanied by an increase in voltage; the arc luminosity appeared as an irregular luminous spot with a clear tendency to expand outward. During the arc expansion stage (Stage II), the current and voltage fluctuated violently, and the arc rapidly spread outward under the combined effects of electric-field forcing and thermal diffusion, with the arc column evolving from an irregular bright patch into an elliptical or columnar shape. During the steady-arcing stage (Stage III), the current and voltage fluctuations became relatively stable; the arc column was stretched and twisted, and a more pronounced arc-root migration behavior on the cathode surface was observed. During the arc extinction stage (Stage IV), continued stretching of the arc column led to an increase in the sustaining voltage due to a voltage jump, accompanied by a reduction in plasma density; the luminous core contracted, whereas the peripheral luminous area expanded and the edge exhibited a bluish-green appearance, indicating substantial heat release and the generation of abundant carbon-containing radicals, until the arc luminosity weakened and finally extinguished.

Overall, as pO_2_ increased, the arc morphology transitioned from a “slender column with intermittent outward spreading” to a “thicker column with a more continuous luminous region.” This trend was particularly evident in the expansion extent of the luminous zone and the tortuous evolution of the arc column during Stage II to Stage III. The observation is consistent with recent simulation studies reporting that arc morphology and motion are governed by different dominant mechanisms across development stages, and that the violent-oscillation stage is driven by complex coupling between electromagnetic forces and thermal buoyancy [31]. In comparison, under the extremely low-oxygen condition (6 vol%), the arc exhibited overall weaker luminosity and reduced column swinging. Under the 21 vol% condition, the arc-initiation moment showed a larger and brighter high-luminance region, yet tail-end collapse of the luminous zone and arc-root hopping occurred more readily. This behavior suggests that arc sustaining is influenced by both oxygen-involved plasma processes and electrode-surface oxidation. As an electronegative gas, oxygen enhances electron attachment and energy dissipation [32], which increases the effective resistance of the discharge channel and promotes an earlier transition into Stage IV after stretching. Meanwhile, faster oxide-film formation on the copper surface under near-ambient oxygen, together with thermal shock, induces time-varying surface conductivity and destabilizes arc-root attachment, resulting in more frequent arc-root migration and stronger arc-column oscillation.

**Figure 4:**
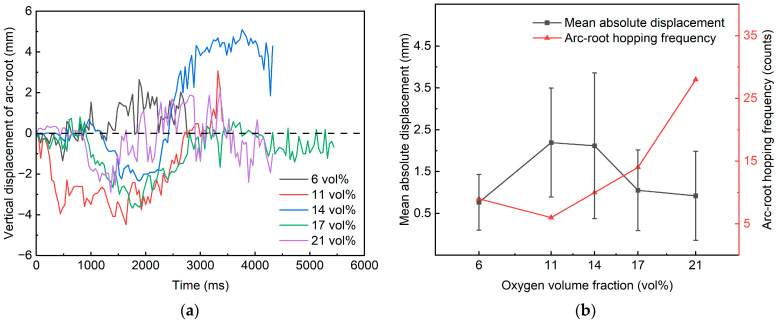
Differences in cathode arc-root motion among the test cases. (**a**) Time-varying curve of arc-root vertical drift; (**b**) Mean absolute displacement, SD, and hopping frequency of the arc-root.

Furthermore, to quantitatively characterize arc-root migration, a frame-interval image annotation method was employed to extract and compare cathode arc-root trajectories under different pO_2_ test cases. The trajectory extraction was calibrated using a unified baseline and a unified scale, and the frame interval for annotation was fixed at 50 frames. Specifically, a unified coordinate system was established with the pre-test electrode contact point as the origin. The horizontal baseline (x-direction) was aligned with the anode copper electrode axis, and the vertical baseline (y-direction) was aligned with the outward normal of the cathode carbon electrode edge. To reduce uncertainty, lateral sliding between the contact pair was neglected during annotation. Only the vertically oriented high-luminance narrow band near the cathode-side electrode boundary during longitudinal offline motion was considered, and the pixel with the maximum brightness was taken as an approximate arc-root location. To quantify arc-root migration intensity and dispersion, the mean absolute value of the arc-root vertical displacement was used to represent the average migration intensity, and the absolute standard deviation (SD) was used to characterize drift dispersion. In addition, to identify arc-root hopping during arcing, the absolute displacement increment between adjacent samples was adopted as the criterion for discrete hopping events, with a hopping threshold of 1 mm. Figure 4 illustrates the differences in cathode arc-root motion under identical experimental parameters for the various test cases.

As shown in Figure 4a, the peak-to-peak amplitude of the cathode arc-root vertical drift for all five test cases was on the millimeter scale, approximately 4 to 7 mm. However, Figure 4b reveals case-dependent differences in the arc-root drift characteristics and hopping frequency. A combined analysis of Figure 4 indicates that the 11 vol% and 14 vol% cases exhibited the largest mean migration intensities, at 2.193 mm and 2.116 mm, respectively, together with relatively high dispersions (1.302 mm and 1.741 mm), suggesting more evident continuous drift and a wider trajectory band. In contrast, the 21 vol% and 6 vol% cases showed smaller mean absolute values (0.920 mm and 0.764 mm), indicating a more “convergent” overall arc-root location. Notably, the hop count displayed an opposite tendency: the 21 vol% case presented the most frequent hopping, with 28 events, markedly exceeding the other cases and accompanied by a relatively large drift dispersion. These results suggest that under near-ambient oxygen levels, the arc root is more prone to rapid, discrete reattachment among multiple potential attachment sites, i.e., high-frequency, large-step jumps, whereas under intermediate and low oxygen levels, the arc root preferentially migrates via sustained drift, manifested as lower-frequency, smaller-step gradual offsets.

### 3.2. Arc Intensity Characteristics

**Figure 5:**
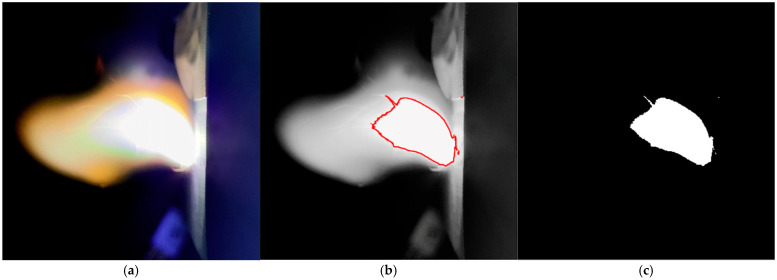
Arc image processing. (**a**) Cropped image (240 px × 240 px); (**b**) image denoising and edge-enhancement, where the red frame marks the target arc region used for threshold tuning and edge extraction; (**c**) binary image.

In this study, image-processing techniques were applied to the arc images captured by the high-speed camera to quantitatively characterize the dynamic evolution of arc intensity under different pO_2_ levels. Although a polarizing filter in front of the lens attenuated part of the harmful radiation, Figure 3 still reveals image noise induced by the enclosed low-light environment and fluctuations of ionized species. To reduce computational cost and uncertainty, the export frame rate of the high-speed imaging system was set to 10 fps. The raw arc images were batch-preprocessed in MATLAB R2024b (MathWorks, Natick, MA, USA) using a consistent pipeline. Specifically, each frame was cropped to 240 px × 240 px and converted to grayscale. Standardized-scale image filtering and edge-enhancement operations were applied to generate high-quality binarized images. To obtain robust segmentation, a preliminary study compared eight candidate combinations of “filtering method–edge enhancement operator.” Manually annotated arc regions were used as reference, and the candidates were evaluated in terms of boundary integrity and the stability of the resulting statistics. Based on these comparisons, wavelet-based denoising combined with a Laplacian enhancement operator was selected to strengthen arc-edge features. The binarization threshold was subsequently tuned according to the grayscale histogram of the processed images, and a unified threshold within the range of 247 to 248 was determined after consistency checks so that a consistent processing scale was applied across all cases. Figure 5 presents a representative image-processing workflow for a selected frame.

**Figure 6:**
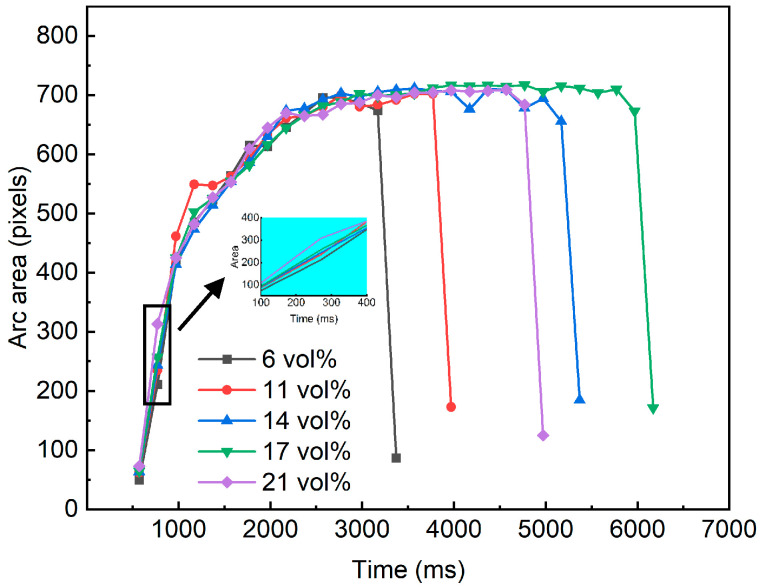
Variation of arc area with pO_2_ levels.

In this study, the high-luminance arc region was identified using a combined strategy of denoising filtering and edge enhancement, as shown by the target region in Figure 5b. The arc area was defined as the pixel count of the binarized arc region in Figure 5c, which was used to dynamically quantify arc intensity. As reported in Ref. [33], pixel-area–based intensity metrics can effectively suppress perturbations caused by image noise and improve the reliability of trend analysis. Figure 6 shows the time evolution of the arc area under different pO_2_ levels.

Overall, the arc intensity in all test cases exhibits a common pattern characterized by “rapid growth, gradual stabilization, and final abrupt decay.” After arc initiation, the area increases rapidly to the order of 10^2^ pixels, after which the growth rate progressively slows and a quasi-plateau forms within approximately 650 to 730. Immediately before extinction, the area drops sharply to around 100 to 200, indicating the collapse of the luminous conduction channel and the onset of the extinction stage. This trend suggests that arc intensity strengthens with the establishment and expansion of the arc column, remains relatively stable in terms of luminous scale during steady arcing, and then undergoes an evident intensity breakdown during extinction.

A further comparison of stage-dependent differences across pO_2_ test cases shows that the area growth in Stage II varies markedly. As illustrated in the zoomed-in view, the arc area at 6 vol% is 211, whereas it reaches 313 at 21 vol%, indicating a stronger early expansion. This quantitative evidence is consistent with the visual observations in Figure 3, suggesting that an increase in pO_2_ facilitates faster establishment of the conductive arc channel shortly after ignition and promotes a larger luminous scale. Meanwhile, other cases also exhibit pronounced area responses in Stage II; for example, at 1170 ms, the area for 11 vol% reaches 544. Together with Figure 4b, these results indicate that during arc-column establishment and expansion, arc intensity is not only governed by pO_2_ but is also jointly modulated by transient arc-root attachment states and arc-column disturbances, which can cause local crossovers among cases within Stage II. After entering Stage III, the arc area gradually converges across cases and fluctuates around a plateau of approximately 680 to 727, reflecting a quasi-steady arc-intensity characteristic during stable arcing. Notably, the 17 vol% case exhibits a slightly larger area in this stage than the other cases, which may be associated with greater energy accumulation during its longer arcing duration, thereby sustaining a larger-scale arc morphology. In Stage IV, arc energy dissipates rapidly and the area decreases sharply; the 6 vol% case completes the decay earliest, whereas the 17 vol% case decays the latest. These observations demonstrate that the area-based visual metric can effectively capture the key dynamic processes of arc evolution from Stage I to Stage IV.

### 3.3. Arc Discharge Characteristics

**Figure 7:**
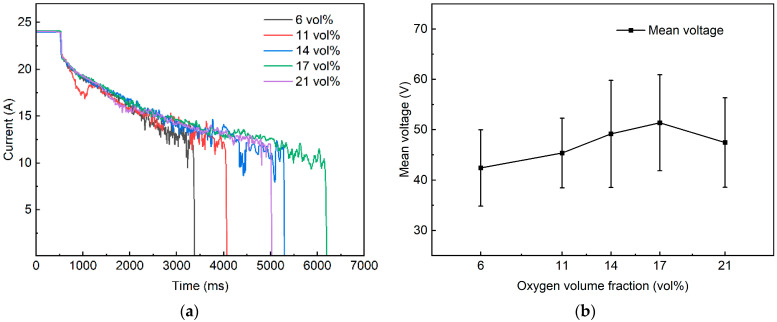
Arc current curve of pantograph–catenary under various pO_2_ levels. (**a**) The results of the first repeated experiments; (**b**) mean voltage and SD of the first repeated experiments.

**Figure A1:**
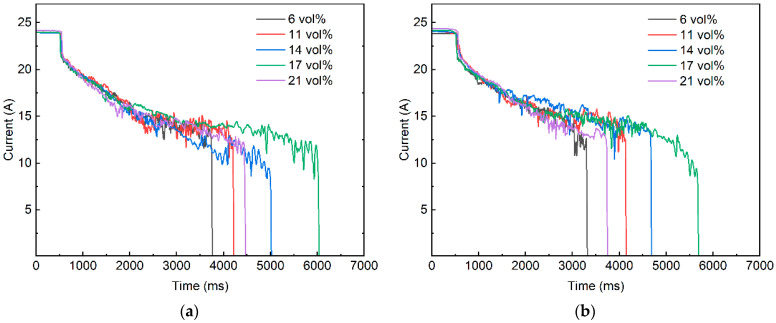
Arc current curve of pantograph–catenary under various pO_2_ levels. (**a**) The results of the second repeated experiments; (**b**) the results of the third repeated experiments.

Monitoring the time-varying current during arcing is a core approach for characterizing current-collection quality and arc dynamic behavior, as its fluctuation features directly reflect the current-carrying capability and the evolutionary trend of arc dynamics. Based on the full-cycle electrical time-series acquired by the DAQ system for the five pO_2_ test cases in Table 3, the raw signals were first converted into physical quantities using the sensor calibration factors listed in Table 2. A steady-conduction segment with the same duration was then retained as a baseline reference. The start and end of the arcing interval were determined according to the four-stage arc-development characteristics, and all subsequent electrical performance metrics were calculated over the extracted arcing interval. Considering that the current and voltage exhibit approximately mirror-symmetric trends, only the current fluctuations are presented to illustrate the trends and case-dependent differences. As shown in Figure 7, the regulation of electrical fluctuations by $x_{O_{2}}$. Figure 7a shows the first repeated test, and Figure 7b presents the mean voltage together with the SD for the corresponding results. The electrical waveforms were time-normalized to a unified duration, and the 0 to 500 ms steady-conduction segment was retained for alignment. Appendix A Figure A1a and Figure A1b present the results of the second and third repeated tests, respectively.

As shown in Figure 7a, the arc-current waveforms under different pO_2_ test cases exhibit a broadly consistent stage-wise evolution, which can be clearly partitioned into four stages in agreement with the stage definitions in Figure 3 and Figure 6. Specifically,

- Stage I (500 to 570 ms). The current departs from the steady sliding-contact conduction state and shows the first pronounced drop to approximately 21 to 22 A. The steep drop slope indicates a strongly transient transition from metallic-contact conduction to plasma-channel conduction. The differences among pO_2_ cases in this stage are minor, suggesting that arc breakdown is primarily dominated by the mechanically induced offline separation process.
- Stage II (570 ms to around 1970 ms). In all cases, the current exhibits a continuous decrease trend superimposed with high-frequency fluctuations of varying intensity, decreasing from ~21 A to ~17 to 18 A. Notably, the 11 vol% case is more prone to larger transient excursions, whereas the 14 vol% and 17 vol% cases show a relatively smoother decrease process. This contrast indicates that the establishment and consolidation of the electrical conduction channel are significantly modulated by pO_2_.
- Stage III (1800 ms to 3330 ms/6120 ms). The current decreased gradually with a progressively reduced decline rate, whereas the arcing duration and the stability of current fluctuations differed markedly among the test cases. The 6 vol% and 11 vol% cases exhibit smaller fluctuation amplitudes and a comparatively gentle evolution, but they decrease earlier and enter Stage IV sooner. In contrast, the 14 vol% and 17 vol% cases present more frequent intermittent “spike-like” disturbances, particularly toward the tail end, with multiple instantaneous drops below 10 A and even quasi-extinction signatures. This behavior implies that intermediate-oxygen levels may introduce a stronger “resistive” contribution to the arc channel, promoting intermittent conduction degradation.
- Stage IV (post-Stage III). A rapid drop of current to 0 A marks arc extinction. The arcing duration shows an evident pO_2_-dependent delay effect. It is noteworthy that the arcing duration at 21 vol% is reduced compared with the intermediate-oxygen cases. This can be rationalized by the higher mean voltage under intermediate pO_2_ levels: as shown in Figure 7b, the 17 vol% case sustains a larger voltage drop during arcing, which facilitates a more persistent balance between energy input and dissipation. Moreover, in conjunction with Figure 4b, when arc-root motion and voltage fluctuations are simultaneously intensified, a chained process of “channel reconstruction–power disturbance” becomes more likely [34], which can shorten the effective arcing duration for the 21 vol%.

The study further revealed a clear correspondence between the current fluctuations in Stage III and arc-root migration. For example, in the 17 vol% case, during approximately 3670 ms to 3840 ms, the current exhibited a short-term fluctuation amplitude of 0.86 A. Concurrent arc images showed a longitudinally twisted and stretched arc root, together with a vertical arc column exerting a pulling driving force on arc-root migration (Figure 3o). This observation indicates that the short-term current undulations in Stage III are not random noise but rather a direct electrical response to arc-root morphological reconstruction and migration. Consistently, a previous study [35] on plasma-arc systems reported that periodic variations in the arc-root attachment location can be “mirror-mapped” by voltage fluctuations.

To quantitatively evaluate the discharge characteristics and current-collection quality of current-carrying off-line arcs under different pO_2_ levels, four arc-performance metrics were introduced in this study: average effective contact resistance, cumulative arc energy, current-carrying efficiency, and current-carrying stability. Considering that the intrinsic resistance of the electrical test circuit and the electrodes may bias the computed results, the resistance of the contact pair was measured three times at 23 °C prior to the experiments, and its mean value accounted for approximately 3.85% of the adjustable load resistance. Therefore, to reduce systematic error and to unify the comparison scale, this contribution was treated as a constant term and removed via differential correction, while dynamic resistance correction associated with temperature rise was not introduced at this stage. The defined average effective contact resistance essentially represents the time-averaged equivalent resistance of the discharge channel, incorporating the combined contributions of the arc column, arc-root region, and interface-related voltage drops, and it is used to quantify the channel’s sustaining conductivity and its resistive character. Based on this, the corrected expression is given as:

(6) $$R_{c}=\frac{1}{n}\sum_{i}^{n}\frac{U_{i}}{I_{i}}-R_{e},$$

Here, $U_{i}$ and $I_{i}$ are the instantaneous sampled voltage and current values within the arcing interval, respectively; *n* is the number of samples; and $R_{e}$ is the mean resistance of the contact pair, which was treated as a constant. The cumulative arc energy was used to characterize the thermal input intensity imposed on the contact pair materials during arcing, and the corrected formulation accounting for Joule heating in the electrodes is given by:

(7) $$E=\int UI-I^{2}R_{e}dt,$$

The integration interval covered the entire arcing duration, where U denotes the arc voltage drop and I denotes the circuit current during arcing. It should be noted that energy integration is sensitive to the time step, and all calculations were verified using a unified sampling scale [36]. To fully account for current fluctuation characteristics and to faithfully reflect the effective energy-transfer capability of the current during arcing, the current-carrying efficiency was defined as:

(8) $$\eta =\frac{I_{rms}}{I_{0}}\times 100\%$$

Here, $I_{rms}$ is the root-mean-square (RMS) value of the arcing current, and $I_{0}$ is the reference current measured after 30 s of steady conduction. $I_{0}$ was taken as the mean of three repeated measurements to mitigate the influence of occasional fluctuations and inherent circuit-resistance bias on the efficiency evaluation. The current-carrying stability was expressed by a normalized fluctuation coefficient to characterize the steadiness and anti-fluctuation capability of current transmission during arcing, and it can be defined as:

(9) $$\delta =\frac{\sqrt{\frac{1}{n}\sum_{i=1}^{n}{\left(I_{i}-\bar{I}\right)}^{2}}}{\bar{I}}\times 100\%,\;$$

**Figure 8:**
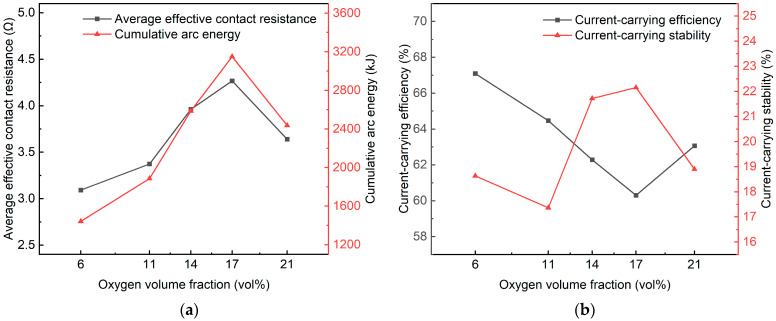
Arc evaluation quality affected by various pO_2_ levels with the test parameters in Table 4. (**a**) Average effective contact resistance and Cumulative arc energy; (**b**) current-carrying efficiency and Current-carrying stability.

In this equation, $\bar{I}$ is the mean current over the arcing interval, and δ is a dimensionless parameter [37]. Figure 8 shows, for the first experimental series, the trends of the arc-performance metrics as a function of $x_{O_{2}}$. Table 5 reports the mean ± SD over three repeated tests (*n* = 3).

Combining the trend lines in Figure 8 with the statistics in Table 5, we found that when the $x_{O_{2}}$ increased from 6 to 17 vol%, three of the four metrics exhibited monotonic trends, except for the stability index. Specifically, the $R_{c}$ increased from 2.901 Ω to 3.820 Ω, and the E increased synchronously from 1663.237 kJ to a peak of 3742.861 kJ at 17 vol%. Meanwhile, the η continuously decreased from 61.749% to 56.070%. In contrast, the δ showed an oscillatory variation, rising from 16.729% to a maximum of 20.306% at 14 vol% and then slightly decreasing to 19.030% at 17 vol%. These results indicate that increasing pO_2_ can markedly deteriorate arc-plasma stability, impair current transfer efficiency, and intensify energy dissipation—features consistent with an enhanced “resistive character” of the arc [38]. Notably, when the $x_{O_{2}}$ further increased to 21 vol%, all metrics showed a partial rebound, exhibiting a non-monotonic response. This non-monotonicity was closely linked to the arcing duration, suggesting a potential “detrimental intermediate-oxygen window” for arc discharge. This window is located approximately around 14 to 17 vol%, where stronger current fluctuations and unstable discharge were induced, accompanied by higher arc voltage and stronger energy input, thereby implying a larger potential thermal-erosion driving force. In contrast, the low-oxygen and near-ambient oxygen regimes can, to some extent, mitigate arcing energy and improve current-transfer performance, with the best behavior observed near the 6 to 11 vol% window.

Unlike the Paschen-curve effect, which primarily concerns Stage I, the instability observed in this study occurred predominantly during Stage II to Stage III. In these stages, the case-dependent fluctuations of the performance metrics indicate substantial differences in the enhanced effective resistance of the discharge channel. We consider this behavior to originate from the coupled regulation of arc-plasma properties, contact-surface chemical reactions, and arc-root dynamics by pO_2_. In light of recent related studies, the underlying mechanisms can be interpreted as follows. As a strongly electronegative gas, oxygen significantly affects electron attachment and ionization, thereby reshaping the electron density and energy distribution of the plasma [39]. Specifically:

- Low $x_{O_{2}}$ (6 to 11 vol%): the arc plasma is dominated by electrons and metal ions evaporated from the contact surface. Under low oxygen availability, electronegative attachment is weak and the probability of electron–oxygen collisions is reduced, leading to a higher effective electron mobility and hence a relatively high arc-column conductivity. Figure 4 further indicates that the arc root is more stably attached to the electrode surface under low $x_{O_{2}}$, with less detachment and hopping, thereby promoting a more continuous current-conduction channel.
- Intermediate $x_{O_{2}}$ (14 to 17 vol%): oxygen dissociation at high temperature produces oxygen-related ions and reactive species, increasing the frequency of electron–oxygen collisions and attachment processes. This reduces the effective electron mobility and weakens the electrical conductivity of the discharge channel, while simultaneously promoting local contraction and re-expansion of the channel, which can trigger instability. In addition, intensified oxidation promotes the formation of a thicker and spatially non-uniform oxide film on the contact surface. The high resistivity and discontinuity of this film can substantially increase the contact resistance and reduce the effective conducting area, thereby hindering efficient current transfer [40].
- Near-ambient $x_{O_{2}}$ (21 vol%): a richer population of reactive oxygen species is generated and interfacial oxidation is strengthened, which can partially suppress melting and evaporation of the electrode substrate [41]. This may reduce collision-related losses associated with excessive metal vapor and thereby slightly improve the effective conductivity of the discharge channel. Meanwhile, sufficient oxidation tends to make the surface oxide film more uniform and compact, which can improve the effective conducting area and stabilize the channel. However, spallation of oxide particles can create local reattachment sites and promote frequent arc-root hopping, consistent with the observations in Figure 4b.

This phenomenon may also be consistent with the hypothesis that the effective conductivity of the arc channel exhibits a local minimum within an intermediate oxygen range. However, verifying such a minimum would require dedicated plasma diagnostics, which is beyond the scope of the present work and will be pursued in future studies.

### 3.4. Erosion Characteristics of Carbon Electrodes

**Figure 9:**
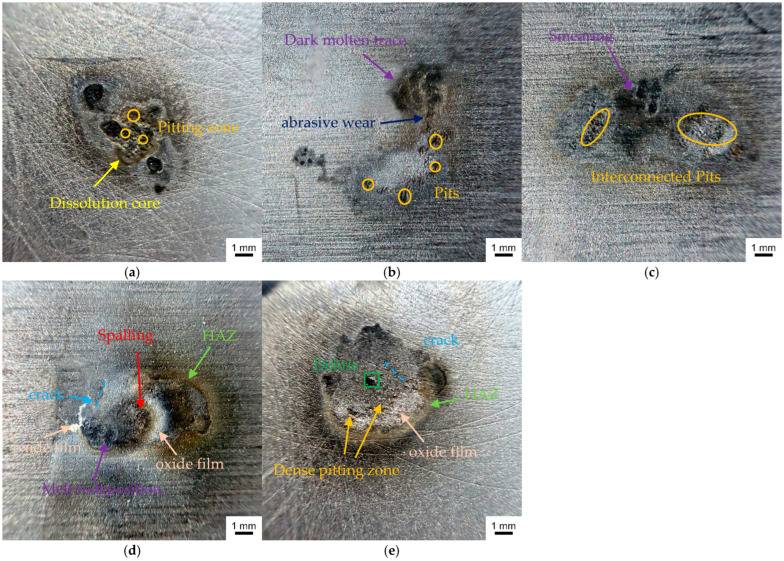
Image of the arc ablated traces. (**a**) 6 vol%; (**b**) 11 vol%; (**c**) 14 vol%; (**d**) 17 vol%; (**e**) 21 vol%.

To qualitatively describe the electrode erosion characteristics under different pO_2_ test cases, macro-scale close-up observations were performed on the arc-eroded surfaces of the carbon plates, as shown in Figure 9. A set of common erosion features can be identified across all cases: the damage is characterized by non-uniform, composite ablation governed by arc-root attachment followed by migration, and is accompanied by prominent point-like erosion pits, dark trailing soot-like traces, and an annular HAZ. These features are commonly reported signatures of arc erosion for Cu/C contacts in the PCS [42].

Upon careful comparison of the micro-scale erosion morphologies across the pO_2_ test cases (Figure 9), distinct differences in the pO_2_-dependent modulation of carbon-electrode erosion can be identified, as detailed below.

- 6 vol% (Figure 9a): The erosion was dominated by small-scale, discrete ablation holes, most of which clustered around the central region, where a rough central dissolution core with a diameter of approximately 3 mm was formed. This feature suggests that under low oxygen availability, the arc root may exhibit short residence near the center, thereby inducing a current crowding effect and generating a localized high heat-flux impact. Meanwhile, because the arcing duration was relatively short and the energy accumulation was limited, the boundary of the HAZ was more confined, and melt-related traces were relatively mild. Overall, the damage primarily manifested as localized high-temperature dissolution accompanied by limited ablation.
- 11 vol% (Figure 9b): The eroded region was elongated into a distinct band following the zigzag sliding trajectory, with ablation traces extending along the sliding direction. Multiple long-distance, quasi-continuous secondary pitting sites were observed within the band, which is in good agreement with the arc-root drift results annotated in Figure 4a, indicating directionally intensified dynamic migration of the arc root. A stronger dark melt-redeposition trace appeared near the tail of the erosion path. Because the erosion path was stretched, the heat-flux density was spatially dispersed, which partially mitigated localized thermal-stress concentration and resulted in overall milder ablation damage.
- 14 vol% (Figure 9c): The eroded zone became noticeably wider, and a smear-like dark agglomerated region was locally visible, indicating more continuous heat-accumulation traces than those at 6 and 11 vol%. This is likely associated with the extended arcing duration, as prolonged thermal exposure tends to intensify pyrolysis and melt-redeposition of carbon-based materials, thereby producing a more pronounced composite morphology involving smearing and edge trailing [43]. In addition, sustained arc-root drift promotes interconnection of adjacent pits, expanding the damaged area and driving a transition from point-like erosion toward sheet-like ablation.
- 17 vol% (Figure 9d): This case exhibited the most severe erosion damage, characterized by a distinct annular HAZ and a composite damage mode involving extensive ablation accompanied by localized melting and melt redeposition, together with non-uniform oxide accumulation near the boundary of the molten region. A prominent white, crack-like film layer appeared in the left region, which is to some extent consistent with the features of a brittle oxide layer fractured after cyclic thermal shock [44], in sharp contrast to the dark molten region on the right. Local observations also revealed delamination of the carbon matrix, which may be attributed to adhesive wear-induced spallation driven by the combined action of frictional heating during current-carrying sliding and arc Joule heating [43].
- 21 vol% (Figure 9e): The erosion scar tended to be more circular with a well-defined boundary, accompanied by a continuous and relatively uniform annular HAZ. A dense oxide-film accumulation region was observed within the scar, which may be related to material transfer and the formation of Cu-containing composite oxides, which may be associated with oxidation-assisted interfacial reactions, potentially including electrochemical oxidation, under high temperature arcing. In the central region, oxide particulates were also visible, and a relatively high density of pitting sites appeared nearby. This observation suggests that under near-ambient oxygen conditions, the arc root is more prone to hopping among multiple potential attachment sites, thereby producing multi-point transient thermal impacts and may contribute to structural degradation and even honeycomb-like pitting characteristics [45].

In summary, pO_2_ exerts a pronounced regulating effect on arc-induced erosion of the carbon electrode. By modulating the plasma sustaining capability and the arc-root reattachment/motion behavior, pO_2_ couples to the arc-root migration mode, local heat accumulation, and the stability of interfacial reaction films. Consequently, the observed erosion morphologies form a cross-scale, internally consistent evidence chain with the previously discussed arc-dynamic evolution and volt–ampere response characteristics. These findings highlight the environmental sensitivity of PCS off-line arcs to pO_2_ differences and provide experimental evidence and mechanistic insight for current-collection quality assessment, material lifetime prediction, and environment-adaptive PCS design under different altitude-related pO_2_ backgrounds. It should be noted that although this study was conducted under a controlled baseline current, the revealed pO_2_-driven mechanisms regulating channel reconstruction and time-varying arc-root reattachment thresholds, together with the proposed “detrimental intermediate-oxygen window” criterion, are transferable and can provide a mechanistic framework and experimental route for future validation under traction-relevant current levels (e.g., 200 to 800 A).

## 4. Conclusions

This study maintained a constant total pressure in a sealed chamber and employed a gas-mixing approach to equivalently establish different pO_2_ levels, thereby experimentally investigating the dynamic evolution, electrical discharge performance, and carbon-electrode erosion response of current-carrying off-line arcs in the PCS. By combining image-processing–based quantification with voltage–current signal analysis, the influences of pO_2_ on arc-root migration, arc intensity, and current-carrying quality were quantified, and the underlying mechanisms were further corroborated through close-up morphological observations of interfacial reaction films and material damage. By benchmarking against existing studies, the following conclusions were drawn:

1. The arc morphology evolved in a stage-dependent manner, and pO_2_ significantly regulated arc-root attachment behavior. With increasing $x_{O_{2}}$, the arc column transitioned from a narrow and intermittently expanding form to a thicker and more continuous form. Meanwhile, the arc-root migration mode changed from a convergent pattern under low-oxygen conditions to continuous drifting under intermediate-oxygen conditions, and finally to high-frequency discrete hopping under near-ambient oxygen conditions.
2. The evolution of arc intensity exhibited a convergent tendency. Under different pO_2_ levels, the arc area followed a consistent temporal pattern of “rapid increase, plateau-like fluctuation, and abrupt decay to extinction.” Under the influence of long-duration energy accumulation, the 17 vol% case exhibited the largest fluctuation amplitude in arc intensity, indirectly confirming that the arc channel was subject to pronounced Joule-heating-driven thermal expansion and buoyancy effects [46].
3. pO_2_ regulated PCS current-transfer performance in a non-monotonic manner, indicating a potential “detrimental intermediate-oxygen window.” The current waveform generally exhibited an intermittent decay trend, and the current-transfer quality deteriorated as $x_{O_{2}}$ increased, whereas the performance metrics rebounded at 21 vol%. In particular, within the intermediate-oxygen range, the current-carrying efficiency dropped to 56.070% while the fluctuation ratio increased to 20.306% (indicating poorer stability), implying that this range is more prone to channel reconstruction and unstable discharge.
4. The arc-erosion morphology of the carbon electrode was constrained by coupled effects of “dynamic migration, heat accumulation, and atmospheric reactions.” With increasing $x_{O_{2}}$, the damage evolved from a centrally localized ablation scar with limited melting traces at 6 vol%, to band-like and sheet-like expanded damage at 11 to 14 vol%, and further to severe ablation with localized melting accompanied by delamination wear at 17 vol%. Under near-ambient oxygen, the dominant damage mode shifted toward electrochemical oxidative wear, manifested by pitting features accompanied by metallic copper transfer. This trend is consistent with Ref. [47], which reported that humid oxygen environments can induce the formation of hydroxylated copper species and promote the diffusion of copper atoms on metal substrates at room temperature.

Overall, compared with introducing atomically thin coatings such as graphene to suppress interfacial oxidation and achieve long-term protection of PCS components under extreme environments [48], the “detrimental intermediate-oxygen window” identified in this study and its mechanistic evidence provide quantitative support for current-collection risk assessment and maintenance scheduling of uncoated PCS components in engineering practice. Future work will further refine the window boundaries, extend the investigation to AC supply, high-current supply regimes, and coupled pO_2_–airflow-field conditions, and explore synergistic strategies involving materials and operating parameters to mitigate erosion and optimize current-collection quality. In addition, SEM and EDS analyses that are widely used in tribological studies [49] will be incorporated to directly verify oxidation products and Cu transfer, thereby strengthening the mechanistic attribution of arc-induced erosion.

## Figures and Tables

**Table 1:** Physical properties of the contact pair specimens *.

Material Parameters	Cu-CATH-2	Pure Carbon
Hardness (10^7^ N·m^−2^)	96.2	67
Specific Heat Capacity (J·kg^−1^·K^−1^)	379.6	709
Density (g·cm^−3^)	8.9	1.82
Electrical Resistivity (µΩ·m)	0.017	12.01
Thermal conductivity (W·m^−1^·K^−1^)	391	90.4
Bending Strength (MPa)	/	48.1

* The operating temperature of the material is 23 °C.

**Table 2:** Signal acquisition devices, models, and key parameters.

Signal Acquisition Device	Model (Manufacturer, City, Country)	Key Parameters
Hall-effect voltage sensor	CHV-50P (SENSOR Electronics, Beijing, China)	Nominal voltage: 1000 V; error ≤ 0.8%
Hall-effect current sensor	CHB50-SF (SENSOR Electronics, Beijing, China)	Nominal current: 50 A; error ≤ 0.8%
Temperature–humidity sensor	SHT31-ARP-B (Sensirion, Stäfa, Switzerland)	RH error ≤ 2% RH; Temp error ≤ 0.3 °C
Oxygen sensor	LFO2-A1 (Alphasense, Great Notley, UK)	Range: 0 to 30 vol%; error ≤ 0.1%
DAQ device	USB-3123 (Smacq, Beijing, China)	16-RSE/8-DIFF

**Table 3:** Grouping of test cases *.

Group	O_2_ Volumetric Fraction (vol%)	Humidity-Corrected pO_2_ (kPa)	Reference Altitude (m)
G1	21.0 ± 0.1	20.9832	98
G2	17.0 ± 0.1	16.9864	1841
G3	14.0 ± 0.1	13.9887	3382
G4	11.0 ± 0.1	10.9912	5218
G5	6.0 ± 0.1	5.9952	9478

* Experimental temperature: 23 °C; relative humidity: 50% RH; total pressure: 101.325 kPa; pO_2_ was calculated with humidity correction, and the reference altitude is provided only as contextual mapping of the pO_2_ range.

**Table 4:** Experimental parameters.

Temperature (°C)	Humidity (% RH)	Atmospheric Pressure (kPa)	Sliding Speed (mm/s)	Offline Speed (mm/s)
23.0 ± 0.3	50 ± 2	101.325 ± 0.5	10	10

**Table 5:** Results of three repeated tests (mean ± SD).

O_2_ Volumetric Fraction (vol%)	Average Arcing Duration (s)	Average Effective Contact Resistance (Ω)	Cumulative Arc Energy (kJ)	Current-Carrying Efficiency (%)	Current-Carrying Stability (%)
6	2.933 ± 0.241	2.901 ± 0.041	1663.237 ± 166.954	61.749 ± 0.866	16.729 ± 1.102
11	3.577 ± 0.081	3.014 ± 0.211	2086.572 ± 49.417	60.301 ± 1.259	16.202 ± 1.651
14	4.450 ± 0.326	3.722 ± 0.934	2647.827 ± 253.595	57.887 ± 3.356	20.306 ± 5.440
17	5.230 ± 0.346	3.820 ± 0.250	3742.861 ± 223.161	56.070 ± 0.655	19.030 ± 2.223
21	3.847 ± 0.652	3.309 ± 0.231	2308.420 ± 435.661	58.990 ± 1.752	17.963 ± 0.298

## Data Availability

The original contributions presented in the study are included in the article; further inquiries can be directed to the corresponding author.

## Abbreviations

The following abbreviations and nomenclature are used in this manuscript:

PCS	Pantograph-catenary system
MHD	Magnetohydrodynamic
DC	Direct current
AC	Alternating current
Temp	Temperature, °C
RH	Relative humidity, %
pO_2_	Oxygen partial pressure, kPa
$x_{O_{2}}$	Oxygen volumetric fraction, %
DAQ	Data acquisition
AFG	Arc fault generator
PET	Polyethylene terephthalate, the chemical formula is (C_10_H_8_O_4_)_n_
PTFE	Polytetrafluoroethylene, the chemical formula is (C_2_F_4_)_n_
px	Pixels
fps	Frames per second
OCL	Overhead contact line
MFC	Mass flow controller
GC	Gas chromatography
SD	Standard deviation
RMS	Root mean square
HAZ	Heat-affected zone
